# Two-year assessment of the efficacy and safety of sitagliptin in elderly patients with type 2 diabetes: Post hoc analysis of the ASSET-K study

**DOI:** 10.1186/s12902-015-0033-2

**Published:** 2015-07-03

**Authors:** Shinichi Umezawa, Akira Kubota, Hajime Maeda, Akira Kanamori, Kiyokazu Matoba, Yasuyuki Jin, Fuyuki Minagawa, Mitsuo Obana, Kotaro Iemitsu, Shogo Ito, Hikaru Amamiya, Mizuki Kaneshiro, Masahiko Takai, Hideaki Kaneshige, Kazuhiko Hoshino, Masashi Ishikawa, Nobuaki Minami, Tetsuro Takuma, Nobuo Sasai, Sachio Aoyagi, Takehiro Kawata, Atsuko Mokubo, Yukiko Miyairi, Hiroshi Takeda, Shin Honda, Hideo Machimura, Tetsuya Motomiya, Manabu Waseda, Yoshikazu Naka, Yasushi Tanaka, Yasuo Terauchi, Ikuro Matsuba

**Affiliations:** Study Group of the Diabetes Committee, Kanagawa Physicians Association, Yokohama, Japan; Division of Metabolism and Endocrinology, Department of Internal Medicine, St. Marianna University School of Medicine, Kawasaki, Japan; Department of Endocrinology and Diabetes, Yokohama City University Medical Center, Yokohama, Japan

**Keywords:** Type 2 diabetes, Sitagliptin, Elderly patients, Dipeptidyl peptidase 4 inhibitor, Sulfonylurea, Hypoglycemia

## Abstract

**Background:**

There have only been a few reports about use of dipeptidyl peptidase 4 (DPP-4) inhibitors in elderly patients with type 2 diabetes mellitus (T2DM), suggesting that the safety of these agents has not been sufficiently demonstrated. We performed a comparative review of the efficacy and safety of sitagliptin for Japanese patients with T2DM managed in the real-world clinical setting.

**Methods:**

An age-stratified analysis was performed of 831 patients who were treated with sitagliptin for 2 years. Parameters assessed included the hemoglobin A_1c_ (HbA_1c_), body weight, serum creatinine, and adverse events. HbA_1c_ and the incidence of hypoglycemia were also evaluated in patients treated with sitagliptin and a sulfonylurea (SU), who were divided into three age groups (<65 years, 65–74 years, and ≥75 years).

**Results:**

Comparison of glycemic control parameters, laboratory values, and adverse events revealed significant improvement of HbA_1c_, casual postprandial plasma glucose, and fasting plasma glucose in each age group with no change in body weight. Serum creatinine increased significantly in all age groups. Hypoglycemia only occurred in patients who received combined treatment with an SU and sitagliptin, and there was no age-related difference in its incidence.

**Conclusions:**

HbA_1c_ was improved by 2 years of sitagliptin therapy in all three age groups, and age did not seem to influence the incidence of hypoglycemic events. These results confirm the efficacy and safety of sitagliptin in patients ≥ 75 years old, suggesting that it is also useful for treating elderly patients with T2DM.

## Background

Elderly patients with type 2 diabetes mellitus (T2DM) are susceptible to osteoporosis [1] and dementia [2], as well as better-known complications such as diabetic microangiopathy and macroangiopathy, and the mortality rate of elderly patients with diabetes is twice that of non-diabetic elderly persons [3]. To maintain a good quality of life for patients with T2DM and improve their prognosis, it is important to achieve appropriate glycemic control with diet and exercise plus pharmacotherapy as required.

Oral hypoglycemic drugs can cause various adverse reactions, including hypoglycemia, weight gain, fluid retention, cardiac failure, and gastrointestinal symptoms [4–6]. In particular, hypoglycemia is associated with cardiovascular events and cognitive dysfunction and it is known that hypoglycemic episodes can lead to falls and fractures [7]. Elderly patients are more likely to develop hypoglycemia than younger patients when treated with multiple drugs, as well as immediately after discharge from hospital, if they have renal failure, and if their diet is poor, and they are also less likely to detect the onset of hypoglycemia [8, 9]. These differences make treatment of diabetes more difficult in elderly patients, so that careful education and drug selection are required.

Sitagliptin was the first dipeptidyl peptidase 4 (DPP-4) inhibitor to be released for clinical use. It is an oral hypoglycemic drug with a glucose-dependent mechanism of action via glucagon-like peptide 1 and glucose-dependent insulinotropic polypeptide, which makes it less likely to cause hypoglycemia [10, 11]. In addition, sitagliptin does not cause weight gain and is expected to protect the pancreatic beta-cells, suggesting that it is an appropriate drug for elderly patients [12–16]. However, there have only been a few reports about use of DPP-4 inhibitors in elderly patients with T2DM, and the safety of sitagliptin has not been demonstrated sufficiently.

We previously investigated the efficacy and safety of sitagliptin for Japanese T2DM patients treated in the real-world setting [17]. In the present study, we performed a post hoc age-stratified analysis of the subjects who received sitagliptin therapy for 2 years in the previous study to evaluate its efficacy and safety for elderly T2DM patients.

## Methods

We previously performed a multicenter cohort study of Japanese T2DM patients to investigate the efficacy and safety of sitagliptin in the real-world setting [17, 18]. Briefly, 28 hospitals specializing in diabetes and belonging to the Study Group of the Diabetes Committee of the Kanagawa Physicians Association participated in the study, which enrolled patients on diet and exercise therapy as well as patients with poor glycemic control (hemoglobin A_1c_ [HbA_1c_] ≥ 6.9 % and the primary physician considered intervention necessary) despite treatment with hypoglycemic agents. After giving consent to participation, the patients started sitagliptin therapy with or without other hypoglycemic medications. Sitagliptin could be up-titrated or down-titrated from the initial dose (25 or 50 mg), and other T2DM medications with a different mechanism of action could be added at the discretion of the primary physician. A total of 1332 patients were enrolled between 2009 and 2010. This study was performed at 28 clinics specializing in the treatment of diabetes by diabetologists who shared information through periodic study sessions.

Hypoglycemia was diagnosed if HbA_1c_ was ≤ 70 mg/dL or typical hypoglycemic symptoms were confirmed by the primary physician, in conformity with the definition of the American Diabetes Association (ADA) [19]. The goal of treatment was to achieve an HbA_1c_ <6.9 % irrespective of the patient’s age, based on the Japanese guidelines around 2009–2010 when this study was started. While exercising due care to avoid hypoglycemic events, treatment was provided to achieve an HbA_1c_ <6.9 % from the perspective of preventing diabetic complications.

In the present post hoc study, we performed age-stratified analysis by dividing the 831 patients who were treated for 24 consecutive months into three groups: <65 years old, 65–74 years old, and ≥75 years old. The study endpoints included HbA_1c_, casual postprandial plasma glucose [20], body weight, serum creatinine, estimated glomerular filtration rate (eGFR), and adverse events.

We also assessed HbA_1c_ and the incidence of hypoglycemic episodes in patients treated with the combination of sitagliptin and a sulfonylurea (SU). Although there were no definite criteria for SU selection in this study, the recommendation issued by the Committee on Proper Use of Incretins [21] was followed by participating physicians, which meant that the maximum doses of glimepiride, gliclazide, and glibenclamide were 2 mg, 40 mg, and 1.25 mg, respectively.

Results are presented as the mean ± standard deviation. Analysis of variance (ANOVA) was employed to assess differences and the level of significance was set at p < 0.05 (two-tailed). All analyses were performed using SPSS version 19 for Windows.

This study was approved by the Ethics Review Board of the Kanagawa Physicians Association.

## Results

**Fig. 1:**
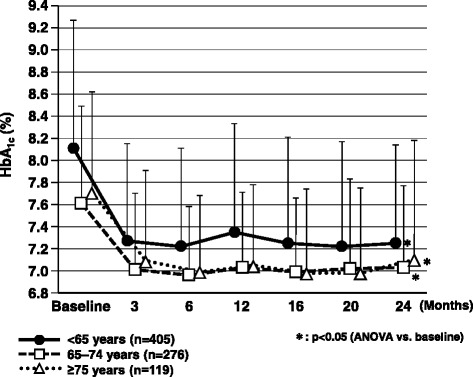
Changes in HbA_1c_ over the 2-year observation period. ANOVA, analysis of variance; HbA_1c_, hemoglobin A_1c_

**Fig. 2:**
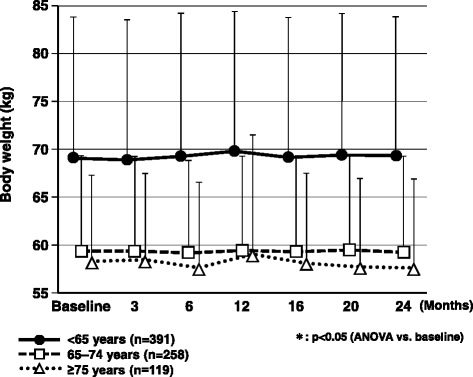
Changes in body weight over the 2-year observation period. ANOVA, analysis of variance

The characteristics of the 1332 patients registered in the ASSET-K study and those of the 831 patients with 2-year data are shown in Table 1. The 831 patients were divided into three age groups for further analysis (Table 2). In patients aged <65 years, HbA_1c_ showed a significant decrease (p < 0.05) from 8.1 ± 1.2 % at the start of add-on treatment with sitagliptin to 7.4 ± 1.0 % at 12 months and 7.3 ± 0.9 % at 24 months. There was also a significant decrease (p < 0.05) in HbA_1c_ in patients aged 65–74 years (7.6 ± 0.9 %, 7.0 ± 0.7 %, and 7.0 ± 0.7 %, respectively). Furthermore, HbA_1c_ decreased significantly (p < 0.05) in patients aged ≥75 years (7.7 ± 0.9 %, 7.1 ± 0.7 %, and 7.1 ± 1.1 %, respectively) (Fig.1). There was no significant change in body weight in any of the age groups (Fig. 2).

**Table 1:** Characteristics of patients registered in ASSET-K and patients with 2-year data

	Overall ASSET-K population	Patients with 2-year data
*n*	1332	831
Age (years)	62.9 ± 11.6	63.3 ± 10.9
Sex (male/female)	751/581	469/362
Duration of diabetes (years)	12.0 ± 8.1	12.3 ± 8.3
Body mass index	24.6 ± 4.3	24.6 ± 4.2
Concomitant medications (%)		
No other OADs	20.0	18.4
1 other OAD	36.0	31.5
2 other OADs	31.0	33.3
≥ 3 other OADs	13.0	16.8

OAD, oral antidiabetic drug

Data are presented as the mean ± standard deviation unless otherwise indicated

**Table 2:** Characteristics of the 831 patients stratified into three age groups

	Age group			P value
	<65 years	65–74 years	≥75 years	
*n* (%)	423 (50.9)	282 (33.9)	126 (15.2)	
Age (years)	54.8 ± 7.8	69.4 ± 2.8	78.2 ± 3.0	<0.0001^*^
Sex (male/female)	271/152	141/141	57/69	<0.0001^†^
Duration of diabetes (years)	9.3 ± 5.9	14.7 ± 8.8	16.8 ± 9.9	<0.0001^*^
Body mass index	25.5 ± 4.6	23.5 ± 3.5	23.7 ± 3.3	<0.0001^*^
Concomitant medications (%)				
Sulfonylureas	61.9	67.4	61.1	0.2763^†^
Biguanides	56.7	50.0	34.9	<0.0001^†^
Thiazolidinediones	27.2	22.3	22.2	0.2764^†^
Alpha-glucosidase inhibitors	9.2	8.9	11.9	0.5763^†^

Data are presented as the mean ± standard deviation unless otherwise indicated

^*^One-way analysis of variance, ^†^Fisher’s exact test

**Fig. 3:**
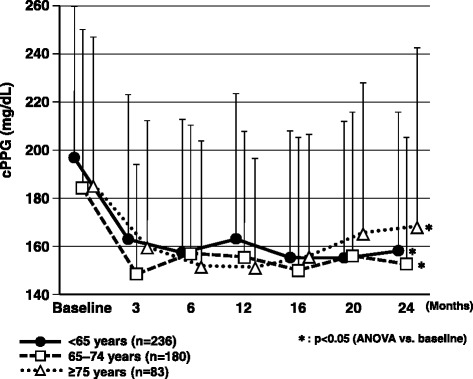
Changes in casual postprandial plasma glucose over the 2-year observation period. ANOVA, analysis of variance; cPPG, casual postprandial plasma glucose

**Fig. 4:**
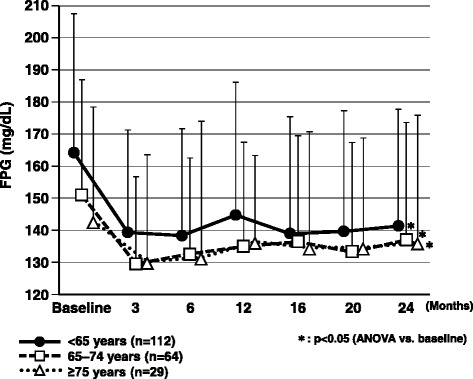
Changes in fasting plasma glucose over the 2-year observation period. ANOVA, analysis of variance; FPG, fasting plasma glucose

Fasting and casual postprandial glucose levels showed a significant decrease at 12 and 24 months compared with the start of add-on sitagliptin therapy in all age groups (p < 0.05) (Figs. 3 and 4).

**Fig. 5:**
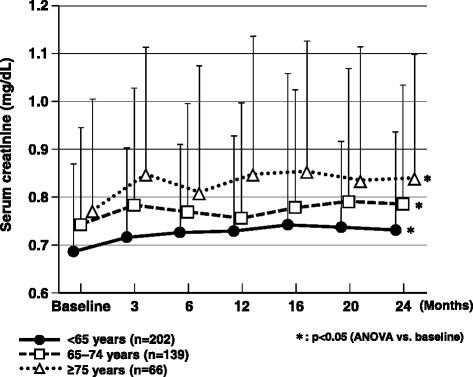
Changes in serum creatinine over the 2-year observation period. ANOVA, analysis of variance

Assessment of renal function showed that serum creatinine was significantly increased at 24 months in all age groups (Fig. 5).

Adverse events reported in at least 2 patients included hypoglycemia (n = 58, confirmed by the primary physician with reference to the ADA definition), constipation (n = 8), rash (n = 5), diarrhea (n = 2), edema (n = 2), and nausea (n = 2). Pancreatitis was suspected in a 42-year-old man due to elevation of amylase, which improved after discontinuation of sitagliptin.

**Fig. 6:**
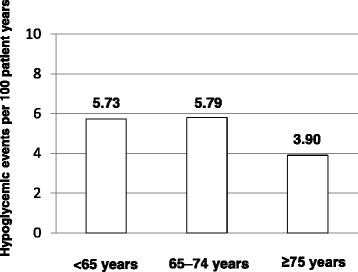
Hypoglycemia in patients receiving sitagliptin plus SU therapy during the 2-year observation period

The 58 patients with hypoglycemia were all receiving sitagliptin combined with an SU drug. Therefore, we analyzed all of the patients treated with sitagliptin plus an SU (529 out of 831 patients) (Table 3). There was no difference in the changes in HbA_1c_ during the 2-year study period between the whole patient population and those treated with sitagliptin plus an SU (Table 4). When the 529 patients were analyzed further and divided into three age groups, the incidence of hypoglycemia was 5.73 events per 100 patients years in patients aged <65 years, 5.79 events per 100 patients years in those aged 65–74 years, and 3.90 events per 100 patients years in those aged ≥75 years (Fig. 6).

**Table 3:** Characteristics of 529 patients receiving sitagliptin plus SU therapy stratified into three age groups

	Age group			P value
	<65 years	65–74 years	≥75 years	
*n* (%)	262 (49.5)	190 (35.9)	77 (14.6)	
Age (years)	54.9 ± 7.6	69.5 ± 5.7	77.9 ± 2.9	<0.0001^*^
Sex (male/female)	143/119	110/80	43/34	0.7853^†^
Duration of diabetes (years)	10.3 ± 6.0	15.7 ± 9.6	18.6 ± 10.1	<0.0001^*^
Body mass index	25.8 ± 4.7	23.7 ± 3.8	24.4 ± 4.4	<0.0001^*^
Type of SU (%)				
Glimepiride	70.6	75.3	71.4	0.5391^†^
Glibenclamide	17.9	14.7	18.2	0.6295^†^
Gliclazide	11.5	10.0	10.4	0.8803^†^
Dose of SU (mg)				
Glimepiride	1.9 ± 1.3	2.0 ± 7.4	1.6 ± 1.1	0.9354^*^
Glibenclamide	4.7 ± 2.5	4.4 ± 2.9	3.0 ± 2.3	0.0007^*^
Gliclazide	37.7 ± 26.4	35.8 ± 32.2	43.3 ± 21.2	0.2708^*^

SU, sulfonylurea

Data are presented as the mean ± standard deviation unless otherwise indicated

^*^One-way analysis of variance, ^†^Fisher’s exact test

**Table 4:** Changes in HbA_1c_ in each age group

	<65 years			65–74 years			≥75 years		
	Baseline	24 months	P value	Baseline	24 months	P value	Baseline	24 months	P value
HbA_1c_ (%) of patients with 2-year data (n = 831)	8.1 ± 1.2	7.3 ± 0.9	<0.05	7.6 ± 0.9	7.0 ± 0.7	<0.05	7.7 ± 0.9	7.1 ± 1.1	<0.05
HbA_1c_ (%) of patients receiving sitagliptin plus SU (n = 529)	8.2 ± 1.3	7.3 ± 0.9	<0.05	7.6 ± 0.9	7.0 ± 0.8	<0.05	7.8 ± 0.9	7.1 ± 0.8	<0.05

HbA_1c_, hemoglobin A_1c_, SU, sulfonylurea

Data are presented as the mean ± standard deviation unless otherwise indicated

## Discussion

The Diabetes Committee of the Kanagawa Physicians Association conducted the ASSET-K study involving over 1,000 patients [17, 18, 22–25], and concluded that factors influencing the reduction in HbA_1c_ by sitagliptin in patients also taking other oral antidiabetic agents were a high baseline HbA_1c_, short duration of diabetes, and low body mass index at the initiation of sitagliptin therapy. Because insufficient evidence was available regarding the safety of sitagliptin therapy for elderly patients, age-stratified analysis of safety and efficacy was performed in the present study.

Although there was the potential for selection bias when the 1332 patients registered for the study were reduced to 831 patients with 2-year data, we performed analysis of the latter group after confirming that the patient characteristics of both groups were similar. This post hoc analysis of ASSET-K data involving 831 patients with T2DM treated in the real-world clinical setting demonstrated that sitagliptin was both safe and effective for elderly patients, a group for which there is particular concern about adverse reactions. HbA_1c_ was improved by 2 years of sitagliptin therapy in patients aged <65 years, 65–74 years, and 75 years or older, although the magnitude of the HbA_1c_ reduction varied due to heterogeneity of the baseline level. There was no significant change in body weight in any age group and weight remained steady for 24 months. Among patients treated with sitagliptin plus an SU, episodes of hypoglycemia showed a similar incidence in all age groups and did not become more frequent with increasing age. On the other hand, serum creatinine increased significantly and eGFR decreased significantly after the start of add-on sitagliptin therapy. As reported previously, the diuretic effect of glucagon-like peptide 1 (which is increased by sitagliptin) leads to elevation of serum creatinine, as also occurs in patients treated with diuretics [22, 25]. Serum creatinine increased slightly in the early treatment period, but subsequently remained steady, suggesting that deterioration of renal function was not progressive during sitagliptin therapy. There was no marked increase in serum creatinine in patients aged ≥75 years after 2 years of treatment in the present study, which an important finding with regard to the safety of sitagliptin for elderly patients.

A separate age-stratified analysis was also performed in patients using sitagliptin concomitantly with SUs, which showed no age-related differences of efficacy or the incidence of hypoglycemia. After medications that influence incretins became available, frequent hypoglycemic episodes were reported in patients taking these agents concomitantly with SUs, leading the Japan Diabetes Society to issue a statement about appropriate use [21]. In the present study, there was no age-related difference in the incidence of hypoglycemia. This study was conducted by diabetes specialists at diabetes clinics and appropriate SU dosages were tailored for each patient in response to the Japan Diabetes Society statement, which may have reduced the incidence of hypoglycemia in our elderly patients.

When combined with sitagliptin, gliclazide (which does not act on Epac2A) is less likely to cause hypoglycemia than glimepiride or glibenclamide (which act on Epac2A) [26]. In the present study, few patients received gliclazide and the incidence of hypoglycemic events was low, which made it difficult to assess differences among these agents. In addition, the glibenclamide dosage was relatively low in patients aged 75 years or older, possibly because the attending physicians considered it desirable to treat elderly patients with lower doses in order to avoid hypoglycemia.

In the present study, pancreatitis was suspected in a 42-year-old man, but the causal relation with sitagliptin therapy is unclear. Butler et al. suggested that incretin-related drugs might induce pancreatitis and pancreatic carcinoma [27]. However, a pooled analysis of clinical trials involving sitagliptin did not show any increase of these conditions. Likewise, no increase of pancreatitis or pancreatic carcinoma was detected in recent large-scale, placebo-controlled, double-blind trials of DPP-4 inhibitors, including SAVOR-TIMI 53 and EXAMINE. Furthermore, a retrospective cohort study of Japanese diabetic patients suggested that treatment with DPP-4 inhibitors does not increase the risk of acute pancreatitis [28]. The prospective TECOS study is currently examining the risk of pancreatitis and pancreatic carcinoma in patients treated with sitagliptin [29, 30], and the results are expected to provide more insight into this issue.

A recent analysis stratified by renal function was performed in the SAVOR-TIMI 53 Trial, a large-scale clinical study of 16492 patients with T2DM that evaluated the impact of saxagliptin (another DPP-4 inhibitor) on cardiovascular disorders. It was found that the efficacy of saxagliptin did not depend on renal function and the drug improved microalbuminuria compared with placebo in patients who had renal impairment [31]. HOMA-2β, an indicator of pancreatic beta-cell function, decreased in the placebo group, but increased in the saxagliptin group after 2 years of treatment [32]. Many elderly patients show impairment of pancreatic or renal function, but the above-mentioned results suggest that long-term treatment with DPP-4 inhibitors may help to preserve residual function.

This study had several limitations. First, there might have been selection bias since this was a retrospective observational investigation without control groups. Second, we only performed analysis of 831 patients from among those registered, although clinical characteristics did not differ between this subgroup and the whole patient population. Third, we had limited information about changes of concomitant medications during the study period, and there were no restrictions regarding the use of concomitant drugs in daily clinical practice. Finally, hypoglycemia might have been underdiagnosed, particularly asymptomatic hypoglycemia that is not uncommon in elderly patients, because detection is largely dependent on symptoms in daily practice. Thus, our findings should be interpreted while taking these limitations into consideration.

## Conclusions

This post hoc analysis of the ASSET-K study assessed the efficacy and safety of sitagliptin therapy in elderly Japanese patients with T2DM. It demonstrated that sitagliptin improves HbA_1c_ without causing hypoglycemia even in patients aged ≥75 years.

## Abbreviations

ANOVA: Analysis of variance
DPP-4: Dipeptidyl peptidase 4
eGFR: Estimated glomerular filtration rate
HbA_1c_: Hemoglobin A_1c_
SU: Sulfonylurea
T2DM: Type 2 diabetes mellitus
